# Mechanisms and regulation of surface interactions and biofilm formation in *Agrobacterium*

**DOI:** 10.3389/fpls.2014.00176

**Published:** 2014-05-06

**Authors:** Jason E. Heindl, Yi Wang, Brynn C. Heckel, Bitan Mohari, Nathan Feirer, Clay Fuqua

**Affiliations:** Department of Biology, Indiana University, BloomingtonIN, USA

**Keywords:** *Agrobacterium*, attachment, biofilm, cyclic-di-GMP, polarity, motility

## Abstract

For many pathogenic bacteria surface attachment is a required first step during host interactions. Attachment can proceed to invasion of host tissue or cells or to establishment of a multicellular bacterial community known as a biofilm. The transition from a unicellular, often motile, state to a sessile, multicellular, biofilm-associated state is one of the most important developmental decisions for bacteria. *Agrobacterium tumefaciens* genetically transforms plant cells by transfer and integration of a segment of plasmid-encoded transferred DNA (T-DNA) into the host genome, and has also been a valuable tool for plant geneticists. *A. tumefaciens* attaches to and forms a complex biofilm on a variety of biotic and abiotic substrates *in vitro*. Although rarely studied *in situ*, it is hypothesized that the biofilm state plays an important functional role in the ecology of this organism. Surface attachment, motility, and cell division are coordinated through a complex regulatory network that imparts an unexpected asymmetry to the *A. tumefaciens* life cycle. In this review, we describe the mechanisms by which *A. tumefaciens* associates with surfaces, and regulation of this process. We focus on the transition between flagellar-based motility and surface attachment, and on the composition, production, and secretion of multiple extracellular components that contribute to the biofilm matrix. Biofilm formation by *A. tumefaciens* is linked with virulence both mechanistically and through shared regulatory molecules. We detail our current understanding of these and other regulatory schemes, as well as the internal and external (environmental) cues mediating development of the biofilm state, including the second messenger cyclic-di-GMP, nutrient levels, and the role of the plant host in influencing attachment and biofilm formation. *A. tumefaciens* is an important model system contributing to our understanding of developmental transitions, bacterial cell biology, and biofilm formation.

## INTRODUCTION

**FIGURE 1:**
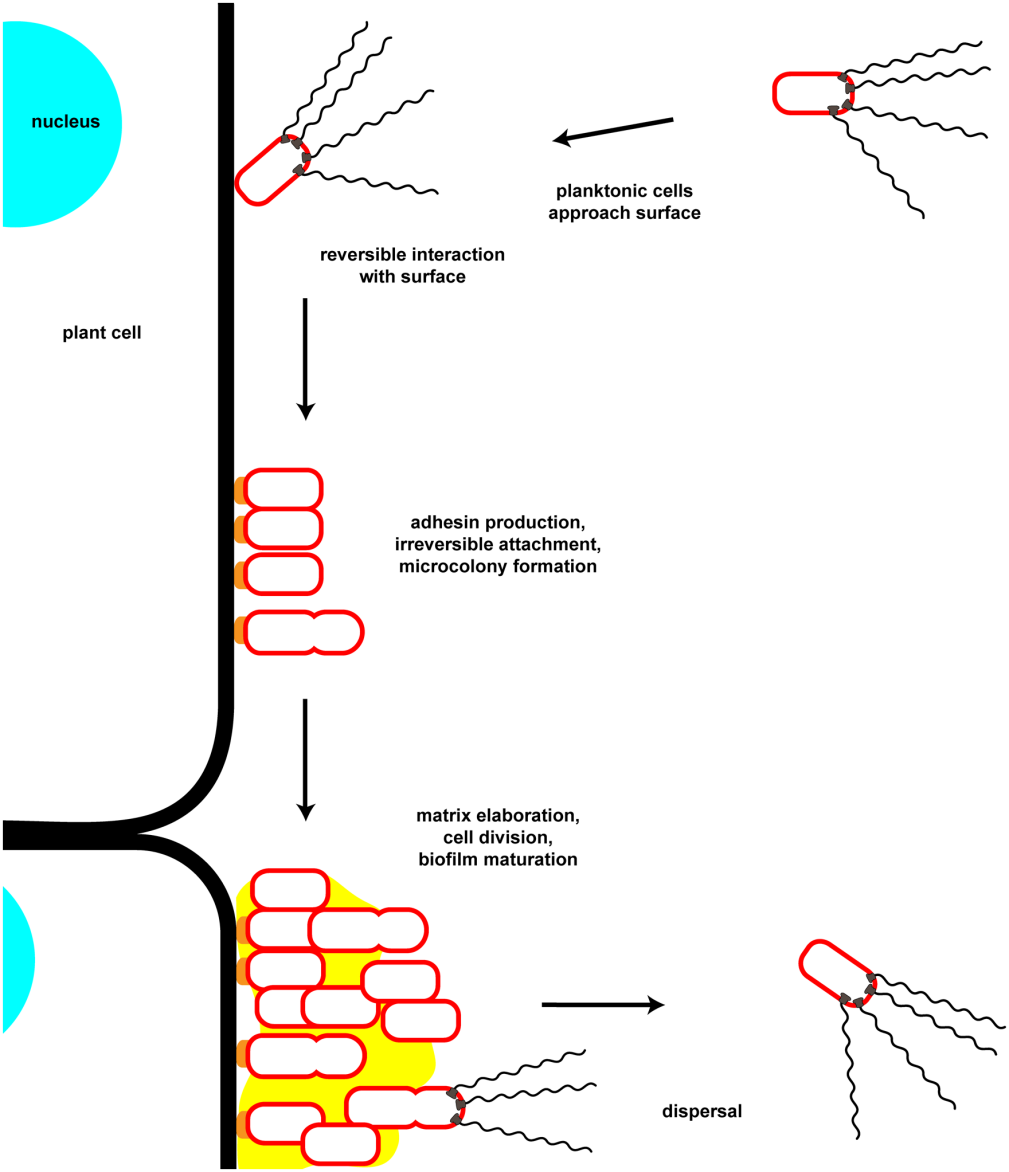
**Key steps in attachment and biofilm formation by *Agrobacterium tumefaciens*.** Motile planktonic cells approach and physically interact with potential attachment substrates. Initial surface interactions are reversible and may depend on physiochemical forces at the interface of the surface with the local medium. Following these initial surface interactions the unipolar polysaccharide (UPP) is secreted by the bacterium at the point of surface contact. This irreversible surface attachment establishes a site for microcolony formation through continued growth and cell division of attached bacteria as well as aggregation of neighboring microcolonies. During and following this period cells secrete matrix components, including cellulose. As the biofilm matures cells may differentiate into various metabolic and reproductive states as the local environment within the biofilm changes. Dispersal from the biofilm may be initiated by an internal developmental cue or by an extracellular factor, as well as through release of motile daughter cells from attached mother cells. Note that in this cartoon only the outer membrane of the Gram-negative cell envelope is depicted.

A biofilm is defined as a multicellular community of one or more microorganisms stably attached to a surface and frequently encased in an extracellular matrix of secreted biopolymers (Costerton et al., 1995). Biofilm formation proceeds from initial contact of an individual bacterium with a surface and reversible attachment, to stable surface association, microcolony formation, biofilm maturation, and to eventual dispersal (Dazzo et al., 1984; **Figure 1**). Biofilms can form on a wide variety of surfaces including living tissues. These multicellular structures and the processes that lead to them are of great interest as they are highly prevalent in the bacterial world, and have profound impacts on society in industrial, medical, and agricultural contexts. The physiology of bacteria within a biofilm is quite distinct from the same cells in a free-swimming, planktonic state. This is best exemplified by the observation that biofilms can manifest dramatically greater resistance to antimicrobial agents, both chemical (e.g., antibiotics, disinfectants) and biological (e.g., viruses, predatory grazing by protists). The control of biofilm growth is therefore quite challenging and a target of significant research. The initial steps of surface attachment that lead to eventual formation of a biofilm are a significant target as control of this step in the process could be used to inhibit the formation of biofilms before they are established, or to promote biofilm formation for beneficial processes. The attachment mechanisms of pathogens to host tissues overlaps with those processes that lead to biofilm formation, and for many pathogens, biofilm formation is an important or requisite component of disease progression. Additionally, the survival of facultative pathogens in environmental reservoirs, such as that for water-borne disease agents, can be dramatically enhanced within biofilms, thereby affecting disease ecology.

*Agrobacterium tumefaciens* is a plant pathogen which is clearly capable of surface colonization and biofilm formation on host tissues, and on abiotic surfaces. This review focuses primarily on the molecular mechanisms by which *A. tumefaciens* initially associates with surfaces and forms a biofilm, as well as the regulation of these mechanisms. Much of the data described below has been determined in the laboratory using the nopaline-type strain *A. tumefaciens* C58. More recent studies on a range of *Agrobacterium* species have revealed similar trends in biofilm formation (Abarca-Grau et al., 2011). It is acknowledged that in many cases the connection between the described attachment and biofilm formation mechanisms and ecological interactions of the bacterium within the rhizosphere remain to be experimentally validated, and much of the relevant environmental context for *A. tumefaciens*, both on and off the plant host, remains poorly understood.

## PHYSICAL INTERACTIONS MEDIATING ATTACHMENT

The first step in attachment and biofilm formation is arrival at and interaction with an appropriate substrate (**Figure 1**). In the rhizosphere this step is frequently mediated by chemotaxis-directed swimming motility as bacteria are attracted toward plant exudates. For many species flagella may also serve as adhesins and there is increasing evidence that inhibition of flagellar rotation, as happens when motile bacteria abut a solid surface, stimulates adhesin production. Active motility may also be required to overcome physiochemical forces at the substrate interface. Additional motility mechanisms as well as multiple adhesin molecules, including pili and various exopolysaccharides, also participate in attachment and biofilm formation.

### FLAGELLUM-DEPENDENT MOTILITY AND ATTACHMENT

There are various forms of motility observed among bacteria, all of which serve to transport bacteria, individually or collectively, through a porous or liquid environment or across a surface (Jarrell and McBride, 2008). These include flagellum-dependent swimming and swarming motility, and flagellum-independent twitching, sliding, and gliding motilities. The particular form of motility used by an individual bacterium is context-dependent and bacteria frequently possess multiple means of locomotion. *A. tumefaciens* is thought to utilize only flagellum-dependent swimming motility (Loake et al., 1988; Shaw et al., 1991; Merritt et al., 2007). Although surfactant production and swarming motility has been observed in the related species *A. vitis* this mode of motility has not yet been described for *A. tumefaciens* (Sule et al., 2009). As with many motile bacteria, in aqueous environments *A. tumefaciens* moves in a series of straight runs, with periodic redirections or tumbles. Directed movement, either toward or away from chemical and physical stimuli, functions by biasing the frequency of tumbles.

*Agrobacterium tumefaciens* typically has a sparse tuft of four to six flagellar filaments, sometimes described as a circumthecal arrangement (Loake et al., 1988; Shaw et al., 1991). Flagellum assembly occurs as a highly regulated process in which a master regulator(s) controls flagellar gene expression. Subsequent regulatory switches drive stepwise expression of subsets of these genes in coordination with different assembly intermediates including the basal body, the hook, and then the flagellum filament. As with several rhizobia, the master regulators of flagellar gene expression in *A. tumefaciens* are called VisN and VisR (Vital for swimming), transcription factors in the LuxR–FixJ superfamily (Sourjik et al., 2000; Tambalo et al., 2010; Xu et al., 2013). VisN and VisR are thought to function in a heterocomplex, and are required for expression of virtually all genes involved in motility. This control is, however, indirect, as VisNR primarily activate expression of another transcription factor called Rem (named in *Sinorhizobium meliloti* for Regulator of exponential growth motility), an OmpR-type two-component response regulator with no obvious partner sensor kinase. Rem is thought to directly activate transcription of the flagellar genes. As will be discussed in more detail in subsequent sections, VisNR also regulate biofilm formation, conversely with flagellar gene expression and independently of Rem, with a profound impact on the process of attachment (Xu et al., 2013).

Motility and chemotaxis play an important role in *A. tumefaciens* attachment, biofilm formation, and virulence. In the rhizosphere, *A. tumefaciens* senses and responds directly to plant exudates, chemotaxing toward plant wounds and inducing virulence gene expression (Loake et al., 1988; Shaw et al., 1988, 1991; Hawes and Smith, 1989; Shaw, 1991). Initial suggestions that flagellar-based motility may influence attachment were based on a set of transposon mutants that lost sensitivity to the flagellum-specific phage GS2 and GS6 (Douglas et al., 1982). The attachment defect in these strains, however, was later linked to pleiotropic effects caused by lesions in *chvA* or *chvB*, genes responsible for generation of β-1,2-glucans (Douglas et al., 1985). Furthermore, *chvAB* mutant strains are virulent when inoculated into plant wounds (Bradley et al., 1984). It was later shown that a putative “bald” strain of *A. tumefaciens*, engineered with disruptions in three flagellin genes (the fourth flagellin gene *flaD* was not known at that time) and microscopically devoid of flagella, was moderately reduced in virulence (Chesnokova et al., 1997). Direct experimental evidence that both chemotaxis and flagellar-based motility affect attachment and biofilm formation was provided by comparisons of defined *A. tumefaciens* mutants with either no flagella, unpowered flagella, or impaired chemotaxis. Deletion of *flgE*, encoding the flagellar hook protein FlgE, generated aflagellate, non-motile bacteria while deletion of *motA*, encoding one of the main components of the flagellar motor, resulted in non-motile cells with unpowered flagella. Aside from their lack of motility, both strains were markedly reduced in both attachment and biofilm formation on abiotic surfaces under static conditions (Merritt et al., 2007). Remarkably, under conditions of constant media flow the Δ*flgE* mutant was increased in attachment and biofilm formation relative to wild-type whereas the Δ*motA* mutant remained impaired. This result suggests that in *A. tumefaciens*, the flagellar filament is not required for attachment and is unlikely to function as an adhesin (Smit et al., 1989a). Rather, active rotation of the flagellar motor is required for both efficient attachment and biofilm formation. Increased rates of attachment and more robust biofilm generation by the Δ*flgE* mutant in a flowing environment might be explained by reduced rates of dispersal from established microcolonies and the biofilm surface.

Chemotaxis mutants, generated by deletion of either the entire chemotaxis operon or the chemotaxis sensor kinase CheA, do not tumble and are impaired for swimming as measured on motility agar plates, a standard laboratory assay for motility (Wright et al., 1998; Merritt et al., 2007). These chemotaxis mutants also manifest significant biofilm deficiencies under both static and flow conditions. By selecting for spontaneous mutants of the Δ*cheA* mutant with increased swimming motility in motility agar, Che^-^ mutation suppressors, or *cms* mutants, were isolated. These *cms* mutants exhibited increased swimming motility on motility agar compared to their parent chemotaxis mutants and were restored for tumbling. Although they improved migration through swim agar, the *cms* mutants remained compromised in attachment and biofilm formation (Merritt et al., 2007). Ectopic expression of a plasmid-borne wild-type *cheA* allele enhanced motility in swim agar but did not correct the attachment deficiency. The improved migration of the *cms* mutants in motility agar in the absence of true chemotaxis resembles the phenomenon known as pseudotaxis (Ames et al., 1996). Pseudotaxis has been described in several systems, including *Escherichia coli* and *Salmonella enterica*, with spontaneous suppressors of chemotaxis mutants developing mutations in flagellar switch genes that lead to increased tumbling rates (Parkinson et al., 1983; Wolfe and Berg, 1989; Magariyama et al., 1990; Sockett et al., 1992; Togashi et al., 1997). The *A. tumefaciens cms* mutants restore tumbling as well, but the basis for their attachment and biofilm deficiencies remains to be elucidated.

### Ctp COMMON PILI AND REVERSIBLE ATTACHMENT

Once the bacterial cell is delivered to a surface via motility or passively by flow, it must initiate physical contact with the substratum. This is often mediated by hair-like extracellular cell surface appendages called pili (or fimbriae) that can function in cell–cell or cell–surface adhesion. Pili in Gram-negative bacteria may be divided into several categories according to their ultrastructure, protein composition, genetic determinants, and mechanism of assembly. These include the type I pili assembled by the chaperone/usher secretion system, the type IV pili assembled by dedicated machinery related to type II secretion systems, and conjugal pili assembled by type IV secretion systems (unrelated to type IV pili; Thanassi et al., 2012). The *A. tumefaciens* genome encodes at least four potential pili. These are the well-studied *virB* T -pilus associated with T-DNA transfer, conjugal pili associated with both pTi and pAt plasmids, and a locus with homology to the type IVb Tad system from *Aggregatibacter actinomycetemcomitans* (Wood et al., 2001). Of these systems, only the type IVb pilus appears to play a role in attachment and biofilm formation by *A. tumefaciens*.

Type IV pili are widespread among diverse bacteria. They are common among Gram-negative species or proteobacteria such as enteropathogenic and enterohemorrhagic *E. coli*, *Legionella pneumophila*, *Neisseria gonorrhoeae*, and *Vibrio cholerae* (Strom and Lory, 1993; Craig et al., 2004; Craig and Li, 2008). Type IV pili are generally 6–9 nm wide, composed primarily of one major pilin subunit, and often aggregate laterally to form bundles. In many species cycles of extension and retraction of type IV pili generate a significant mechanical force, enabling a variety of non-adhesive functions including twitching motility, DNA uptake during transformation, and phage infection (Mattick, 2002). Type IV major pilin subunits are usually synthesized as a prepilin monomer with an N-terminal hydrophilic leader peptide. Type IV pili are grouped into two categories: type IVa pili, whose pilin subunits have short leader peptides (<10 residues) and are 150–160 residues long, and type IVb pili, whose pilin subunits have longer leader peptides (15–30 residues) and are either long (180–200 residues) or are very short (40–50 residues; Mattick, 2002; Thanassi et al., 2012).

The Tad (tight adhesion) system was originally discovered in the periodontal pathogen *Aggregatibacter actinomycetemcomitans* where it mediates attachment and biofilm formation in the oral cavity and may contribute to infective carditis caused by this organism (Scannapieco et al., 1983, 1987; Rosan et al., 1988; Tomich et al., 2007). More recently homologous systems have been identified in many bacterial and archaeal species, including *Yersinia pestis*, *V. cholerae*, *Mycobacterium tuberculosis*, and *Pseudomonas aeruginosa* (Kachlany et al., 2000; Tomich et al., 2007). The *tad* locus is responsible for biogenesis of adhesive Flp (fimbrial low-molecular-weight protein) pili, within the type IVb pilus subclass, which are often involved in biofilm formation and pathogenesis. Several Alphaproteobacteria closely related to *A. tumefaciens*, including *Caulobacter crescentus* and *S. meliloti*, also encode genes homologous to the *Aggregatibacter actinomycetemcomitans tad* locus (Skerker and Shapiro, 2000; Fields et al., 2012). In *C. crescentus* this locus, the *Caulobacter* pilus assembly locus (*Cpa*), is responsible for generating developmentally regulated polar pili that are required for surface interactions and attachment (Skerker and Shapiro, 2000; Bodenmiller et al., 2004; Li et al., 2012). The *A. tumefaciens* genome sequence revealed the *ctpABCDEFGHI* (cluster of type IV pili) locus homologous to the *Aggregatibacter actinomycetemcomitans tad* locus (Wood et al., 2001; Tomich et al., 2007). For the *A. tumefaciens* locus, *ctpA* is predicted to encode the major pilin subunit and *ctpB* the prepilin peptidase that cleaves the leader peptide for pilin maturation. The remaining *ctp* genes encode components of the biosynthetic machinery and related secretion apparatus. Transmission electron microscopy (TEM) of *A. tumefaciens* reveals the presence of thin filaments, significantly thinner than flagella, arranged around the cell surface and frequently shed into the external milieu. These filaments are absent in TEM images of mutant strains deleted for *ctp* genes suggesting that these genes encode Flp-type pili (Wang et al., 2014). As in *C. crescentus*, the Ctp pilus, or a component thereof, may be involved in attachment and subsequent biofilm formation. Mutations in *ctpA*, *ctpB*, or *ctpG* (a predicted ATPase responsible for energizing pilus biogenesis) result in partial but significant decreases in attachment and biofilm formation, and a notable decrease in reversible surface interaction compared to the wild-type strain. Taken together, these results indicate that the *ctp* locus is involved both in pilus assembly, attachment and biofilm formation. Unexpectedly, mature pilin subunits themselves appear to contribute to attachment and biofilm formation, even in mutants for which the Ctp pilus does not assemble (Wang et al., 2014). Modulation of surface interactions by pilin proteins independent of pili has been reported in other bacteria. For example, the minor pilin subunits of *P. aeruginosa*, PilX and PilW, modulate intracellular levels of the second messenger cyclic diguanylate monophosphate (cyclic-di-GMP, or c-di-GMP) and consequently inhibit swarming motility in this pathogen (Kuchma et al., 2012).

### POLAR ATTACHMENT TO SURFACES

At some point weak, reversible surface interactions can transition to more stable associations (**Figure 1**). Several well-studied biofilm-forming bacteria such as *P. aeruginosa* transition from transient interactions in which single cell poles engage the surface, to a longitudinal position (Petrova and Sauer, 2012). This is thought to represent the switch to highly stable, irreversible attachment. Polar surface binding is evident in many micrographs of *A. tumefaciens* associated with plant tissues (Pueppke and Hawes, 1985; Brown et al., 2012), and is consistent on abiotic surfaces in flowing and non-flowing environments, and within complex biofilms (Li et al., 2012; Xu et al., 2012, 2013). It is not clear that polar surface interaction is the only way in which *A. tumefaciens* engages with surfaces, but it is certainly a common mode of interaction. In contrast to the switch from polar to non-polar interactions observed for *P. aeruginosa* and other bacteria, many stably attached *A. tumefaciens* remain associated by a single pole. Recent studies have suggested a model in which during T-DNA transfer to plants *A. tumefaciens* transitions to lengthwise interactions and transfers the DNA via type IV secretion complexes interspersed in an arrayed pattern along the length of the cell (Aguilar et al., 2010, 2011; Cameron et al., 2012). This work uses very high resolution deconvolution microscopy and contradicts previous studies indicating that the type IV secretion complexes localize predominantly to poles (Lai et al., 2000; Atmakuri et al., 2003, 2007; Judd et al., 2005a,b). It is certainly possible that although *A. tumefaciens* might establish stable polar interactions with surfaces, upon induction of the Vir system and initiation of T-DNA transfer to plant cells, it switches to a lengthwise association. Although we observe consistent polar association with both living plants and abiotic surfaces, the two models are not mutually exclusive. Polar attachment is also consistent with the asymmetric budding division of *A. tumefaciens* (described below) where newly born daughter cells are released from the attached mother cell (Brown et al., 2012). The relationship between polar surface binding and the orientation of the *A. tumefaciens* cell during T-DNA transfer has yet to be explained, and new insights may require time lapse analysis of surface binding and T-DNA transfer.

### EXTRUSION OF A UNIPOLAR POLYSACCHARIDE ADHESIN

The stable polar attachment of individual cells to surfaces and to other cells seemed likely to be mediated by adhesin molecules in some manner localized to the cell pole. Unipolar attachment mediated by a polarly localized polysaccharide-containing adhesin is particularly common among Alphaproteobacteria, and is best studied in the Caulobacteraceae and Rhizobiaceae families. Among the stalked members of the Caulobacteraceae this adhesin is called the holdfast and has been extensively studied in *C. crescentus*, in which it is produced at the end of the polar stalk (Poindexter and Cohenbazire, 1964; Poindexter, 1981). In the related *Asticcacaulis biprosthecum*, and *Asticcacaulis excentricus* with non-polar stalks, the holdfast is not localized to the stalk ends, but rather the holdfast localizes to the cell pole (Poindexter and Cohenbazire, 1964; Umbreit and Pate, 1978; Merker and Smit, 1988). In these bacteria, holdfast synthesis and export occurs via a Wzy-type mechanism related to capsule biosynthesis in *E. coli* (Smith et al., 2003; Toh et al., 2008; Cuthbertson et al., 2009). The holdfast of *C. crescentus* is well-characterized in terms of synthesis, export, and physical properties, yet little is known regarding its composition (Tsang et al., 2006; Berne et al., 2013). Based on lectin binding the holdfast is thought to contain *N*-acetylglucosamine residues and is anchored to the cell surface via a functional amyloid protein (Merker and Smit, 1988; Hardy et al., 2010). The strength of this adhesive is remarkable and it has been described as “nature’s strongest glue” (Tsang et al., 2006). Several Rhizobiaceae also attach to surfaces via a polysaccharide adhesin localized to a single cell pole (Dazzo et al., 1984). *Rhizobium leguminosarum*, for example, has a unipolar glucomannan adhesin (Laus et al., 2006). This polysaccharide contains largely glucose and mannose sugar residues, plus detectable amounts of galactose and rhamnose, and is required for specific binding to pea roots, recognized by a lectin produced by peas. Current data show that this unipolar glucomannan interacts directly with a plant lectin rather than acting as a general adhesin. An additional acidic polysaccharide has also been shown to participate in attachment to plastic surfaces and biofilm formation in *R. leguminosarum*, although there is no indication that this polysaccharide is polarly localized (Russo et al., 2006; Williams et al., 2008). More recently a glucomannan-independent acidic polysaccharide-dependent polar attachment has been observed for *R. leguminosarum*, a mode of attachment that is also dependent on the presence of plant arabinogalactan-like glycoproteins (Xie et al., 2012).

The unipolar polysaccharide (UPP) of *A. tumefaciens* is an extracellular polysaccharide with facile similarity to both the *C. crescentus* holdfast and the glucomannan exopolysaccharide of *R. leguminosarum* (Tomlinson and Fuqua, 2009; Xu et al., 2012). Like the holdfast of *C. crescentus* and *Asticcacaulis biprosthecum*, the UPP is produced at a single cell pole upon surface contact (Li et al., 2012; Xu et al., 2012). Wild-type *A. tumefaciens* rarely produces the UPP during planktonic or colony growth (Xu et al., 2013). The *C. crescentus* holdfast is also developmentally regulated and this may be the case as well for the *A. tumefaciens* UPP (Janakiraman and Brun, 1999; Kim et al., 2013). The UPP is known to play an essential role in attachment and biofilm formation on abiotic surfaces, and may also be required for efficient binding to host plants (Xu et al., 2012, 2013). Although it is not yet known how its adhesive strength compares to the *C. crescentus* holdfast, it is clearly an effective cellular adhesin.

Visualization of the UPP was achieved by staining surface-adhered cells with fluorescently labeled wheat germ agglutinin (WGA), an *N*-acetylglucosamine-specific lectin known to label the holdfast of *C. crescentus* (Tomlinson and Fuqua, 2009). Later it was shown that the *N*-acetylgalactosamine-specific lectin *Dolichos bifloris* agglutinin (DBA) similarly labeled a polarly localized structure (Xu et al., 2012). Thus, the UPP is likely to contain at least two sugars, *N*-acetylglucosamine and *N*-acetylgalactosamine. The first gene verified to be required for UPP biosynthesis was *uppE*, a homolog of *C. crescentus hfsE*, the initiating glycosyltransferase for holdfast synthesis. The *uppE* locus was identified in a screen for *A. tumefaciens* mutants that were deficient in attachment and biofilm formation (Xu et al., 2012). It is clear that *uppE* and the surrounding genes comprise an incomplete Wzy-type polysaccharide biosynthesis cluster, *uppABCDEF* (Atu1235–1240), and are orthologous to the genes required for unipolar glucomannan in *R. leguminosarum* (Williams et al., 2008). This suggests that both adhesins may share structural or functional similarities. Nonetheless, the unipolar glucomannan of *R. leguminosarum* and the UPP of *A. tumefaciens* are clearly not identical, perhaps reflecting different host preferences and lifestyles. It is hypothesized that additional genes are involved in UPP biosynthesis as several key functions including a flippase (Wzx) and a polysaccharide polymerase (Wzy) homolog have not yet been identified.

Interestingly, the requirement for *uppE* is conditional. Phosphate limitation abrogates the requirement for *uppE*. Genetic analysis revealed a conditional redundancy for *uppE* and a paralogous initiating glycosyltransferase, Atu0102 (Xu et al., 2012). The *uppE* gene is required for UPP biosynthesis under phosphate-replete conditions whereas *uppE* and Atu0102 function redundantly under conditions of limiting phosphate. The underlying basis for this conditional functional redundancy remains unclear, but may involve the intracellular signal c-di-GMP.

### CONTACT-DEPENDENT ATTACHMENT AND JUST-IN-TIME ADHESIN DEPLOYMENT

Thus far only the general requirement for the UPP in *A. tumefaciens* attachment and biofilm formation has been described. It was also noted that the UPP is not produced by planktonic cells, or cells in colonies. How is such temporal control over UPP synthesis and export achieved? In several genera of Alphaproteobacteria, including *C. crescentus*, *A. tumefaciens*, and *A. biprosthecum*, contact with a solid surface stimulates production of a polar polysaccharide-containing adhesin such as the holdfast and UPP. Biosynthesis and export of this adhesin enables the transition from reversible to irreversible attachment (Li et al., 2012). Surface sensing, and subsequent adhesin production, was demonstrated to be pili- and flagellum-dependent in *C. crescentus*, requiring inhibition of the flagellar motor. The exact molecular mechanism by which inhibition of flagellar rotation regulates adhesin production is not clear. This would not, however, be the first example of the flagellum being used as an environmental sensor. In the pathogenic marine bacterium *V. parahaemolyticus*, it has been shown that the polar flagellum senses surface contact, enabling differentiation of this organism into a swarming motility-competent cell type (McCarter and Silverman, 1990; Gode-Potratz et al., 2011). In *V. cholerae* flagellum interaction with a surface results in a transient loss in membrane potential that ultimately effects the transition to the attached state (Van Dellen et al., 2008). More recently, inhibition of the MotA/MotB stator in *Bacillus subtilis* was demonstrated to effect poly-γ-glutamate (PGA) production, an extracellular capsular polymer (Chan et al., 2014). It is intriguing to imagine that a similar mechanism might extend into the Alphaproteobacteria.

The mechanism of surface sensing and consequent adhesin production in *A. tumefaciens* and *Asticcacaulis biprosthecum* is not known. It is hypothesized that flagellar rotation and pili may participate, as in *C. crescentus*. Of note, and described earlier, polar adhesin production and just-in-time deployment functions normally in non-piliated *A. tumefaciens* mutants (Wang et al., 2014). Importantly, contact-dependent polar adhesin production in *A. tumefaciens* was also shown to efficiently occur on the plant root surface (Li et al., 2012). It is likely that the regulatory signals that direct just-in-time deployment of the *A. tumefaciens* UPP adhesin control additional aspects related to attachment and biofilm formation. Indeed it has been suggested that the elaboration of cellulose fibrils occurs only after the initial attachment process in both *A. tumefaciens* and *R. leguminosarum* (Matthysse et al., 1981; Smit et al., 1987). Just-in-time deployment of the UPP is hypothesized to prevent occlusion of the adhesive by soluble ligands and unproductive autoaggregation of planktonic bacteria, also allowing conservation of resources until the bacterial cell is proximal to a solid surface. As mentioned above and described below, planktonic *A. tumefaciens* cells generally do not generate the polar adhesin unless key regulatory components and signaling circuits are disrupted.

## BIOFILM COMPOSITION

Over time surfaces colonized by irreversibly attached individual *A. tumefaciens* cells may undergo a profound transition to a multicellular state, the biofilm (**Figure 1**). Biofilms comprise a community of bacterial cells attached to a surface and surrounded by a hydrated macromolecular matrix (Costerton et al., 1995). Matrix components may include one or more extracellular polymeric substances, including exopolysaccharides, extracellular DNA (eDNA), and protein components (Flemming and Wingender, 2010). The *A. tumefaciens* genome encodes for production of at least six polysaccharide species, several of which play roles in attachment and biofilm formation. These include the UPP adhesin (described above), cellulose, succinoglycan, cyclic β-1,2-glucans, β-1,3-glucan (curdlan), and outer membrane lipopolysaccharide (LPS). Thus far there are no data suggesting that either eDNA or proteinaceous components are found as structural elements in the matrix of the mature *A. tumefaciens* biofilm. A possible role for a protein adhesin, the so-called rhicadhesin (Rhizobiaceae calcium-binding adhesin) protein has been shown for attachment. The matrix of many bacterial species contains one or more functional amyloid proteins as a structural element, with perhaps the most well-known examples being CsgA (curlin) of *E. coli* and TasA of *B. subtilis* (DePas and Chapman, 2012). Several strains of *A. tumefaciens* and related strains from *R. etli* encode a cluster of genes with homology to the functional amyloid curlin, but these have yet to be assigned any physiological role in these bacteria.

### CELLULOSE

Cellulose is frequently found as a component of the biofilm matrix in many organisms including several members of the Rhizobiaceae (Karatan and Watnick, 2009; Flemming and Wingender, 2010; Bogino et al., 2013). Cellulose, perhaps the most abundant organic polymer on Earth, is produced by nearly all plants and many bacteria, as well as within the animal and fungal kingdoms (Delmer, 1987; Römling, 2002; Matthysse et al., 2004; Sagane et al., 2010). Cellulose is a homopolymer of β-1, 4-linked glucose monomers with individual cellulose fibers consisting of thousands of individual subunits. The mechanism of prokaryotic cellulose biosynthesis has been well-studied in the Alphaproteobacterium *Gluconacetobacter xylinus* (Ross et al., 1987). Homologous systems for cellulose biosynthesis were later found in *A. tumefaciens*, *E. coli*, and *Salmonella enterica*, among others (Amikam and Benziman, 1989; Matthysse et al., 1995b; Zogaj et al., 2001). Prior to identification of synthetic and regulatory genes involved in cellulose production in *A. tumefaciens*, a role for cellulose in attachment to plant surfaces was reported (Matthysse et al., 1981). The production of cellulose by *A. tumefaciens* results in loose aggregation of planktonic cells (flocculation), pellicle formation in static cultures, and loose attachment to surfaces. Although not absolutely required for virulence, cellulose mutants do show a slightly reduced ability to colonize plants and induce tumor formation (Matthysse, 1983). Overproduction of cellulose enhances attachment to plant roots in *A. tumefaciens* (Matthysse et al., 2005). Cellulose synthesis in *A. tumefaciens* requires genes in two operons, *celABCG* and *celDE* (Matthysse, 1995; Matthysse et al., 1995b, 2005). The *celA* gene encodes a protein homologous to the catalytic subunit of cellulose synthase (BcsA) from *G. xylinus*, and contains a PilZ domain at the C-terminus, allowing for potential allosteric regulation via c-di-GMP. CelB homologs are also known to bind c-di-GMP and likely function as regulatory subunits of cellulose synthase. CelC has homology to secreted endoglucanases while *celD* and *celE* are soluble, cytoplasmic components involved in early steps of cellulose polymerization. Several negative and positive regulators of cellulose synthesis have been identified, including CelG and CelI (Matthysse et al., 1995a). Mutations in either *celG* or *celI* results in increased cellulose production, indicating that these gene products encode negative regulators of synthesis. Mutations in the *A. tumefaciens* homologs of *divK* or *pleD* (*celR*) also affect cellulose production (see Coordination of Division and Development; Barnhart et al., 2013, 2014). Similar results have been observed in *R. leguminosarum* (Ausmees et al., 1999). As described below, many regulatory aspects of cellulose synthesis parallel that of UPP regulation, with c-di-GMP being one of the primary regulators.

### ROLE OF OTHER POLYSACCHARIDES IN ATTACHMENT AND BIOFILM FORMATION

As mentioned earlier, aside from the UPP and cellulose, *A. tumefaciens* produces at least three additional exopolysaccharides: succinoglycan, cyclic β-1,2-glucans, and curdlan (Nakanishi et al., 1976; Hisamatsu et al., 1978; Zevenhuizen and Vanneerven, 1983; Karnezis et al., 2003). The major acidic EPS produced by *A. tumefaciens* is succinoglycan, the product of the *exo* genes (Cangelosi et al., 1987). The role of succinoglycan in the biology of *A. tumefaciens* is unclear. Mutants unable to synthesize succinoglycan are fully virulent, efficiently attach to plant surfaces, and are not diminished in biofilm formation (Tomlinson et al., 2010). In contrast, in *S. meliloti* succinoglycan (also called EPS I) is required for biofilm formation and productive interaction with the plant host (Cheng and Walker, 1998; Fujishige et al., 2006). It was recently proposed that the physiochemical properties of succinoglycan contribute to aggregation in *S. meliloti*, and that this may eventually lead to productive biofilm formation (Dorken et al., 2012). It is possible that succinoglycan may play a similar role in some environments for *A. tumefaciens*, although at present there are no supporting data to this effect. In both *A. tumefaciens* and *S. meliloti* succinoglycan synthesis is negatively regulated by a periplasmic protein, ExoR (Chen et al., 2008; Tomlinson et al., 2010). ExoR is itself sensitive to pH and thus it is possible that one function of *A. tumefaciens* succinoglycan is related to acid tolerance (Lu et al., 2012; Wu et al., 2012).

β-1,2-Glucans may be generated in linear or cyclic forms and are synthesized by many rhizobia (Breedveld and Miller, 1994). In *A. tumefaciens* β-1,2-glucans are cyclic, the product of the ChvB synthase (Puvanesarajah et al., 1985; Zorreguieta et al., 1988; Castro et al., 1996). The *chvB* (*ch*romosomal *v*irulence) gene was originally isolated in a transposon screen for mutants unable to attach to plant cells and required for virulence (Douglas et al., 1982). A second locus adjacent to *chvB* also identified in this screen is *chvA*, the product of which is required for export of β-1,2-glucans into the periplasm where they are believed to play a role in osmoadaptation (de Iannino and Ugalde, 1989; O’Connell and Handelsman, 1989). While the genes directing synthesis of cyclic β-1,2-glucans were isolated due to their attachment and virulence phenotypes, a direct role for this polysaccharide species in attachment has not been demonstrated. Rather, impaired osmoregulation within the periplasmic space results in pleiotropic effects on the cell surface, several of which likely contribute to the attachment deficiency (Breedveld and Miller, 1998). As well as being deficient in attachment to plant surfaces, mutants in *chvA* or *chvB* also show a modest decrease in biofilm formation (Xu et al., 2012).

Curdlan is a neutral β-1,3-glucan produced by many bacteria and utilized as a gelling agent in the food industry (McIntosh et al., 2005). While most work on curdlan biosynthesis has been performed in the curdlan-overproducing strain *Agrobacterium* sp. ATCC 31749, genome analysis of *A. tumefaciens* indicates that the curdlan synthesis genes are conserved. Although the regulation of curdlan synthesis in *Agrobacterium* sp. ATCC 31749 shares many features with regulation of other exopolysaccharides, no biological function has been described for this polysaccharide species in *A. tumefaciens* (Ruffing and Chen, 2012). Deletion of *crdS*, encoding the curdlan synthase homolog in *A. tumefaciens* has no effect on attachment and biofilm formation (Xu et al., 2012).

Early work suggested that *A. tumefaciens* LPS was required for attachment to plant surfaces (Lippincott and Lippincott, 1969; Whatley et al., 1976). This work demonstrated inhibition of attachment to wound sites with crude preparations of LPS. It is unclear what other inhibitors may have been present in this preparation. Other than these findings, there are no other data supporting a role for LPS in attachment and biofilm formation, although many of the genes encoding LPS synthesis would be essential, and genetic studies might therefore not reveal a role for this surface polysaccharide. The localization of LPS on the outer leaflet of the outer membrane certainly might impart an influence on surface interactions, and in other bacteria LPS has been demonstrated to impact attachment to surfaces.

### RHICADHESIN AND RAPS

Although the UPP and cellulose are important adhesins mediating attachment and biofilm formation in *A. tumefaciens*, it is possible that additional adhesins may contribute to either process. The activity of these putative adhesins may be discernible only under particular circumstances, indicative of temporal or developmental regulation or a specific plant host interaction. One possible adhesin present in the rhizobia is the calcium-dependent protein rhicadhesin, originally identified in *R. leguminosarum* strain 248 (Smit et al., 1987). Under calcium-limiting conditions *R. leguminosarum* was reduced both in its ability to agglutinate to glass and to attach to pea root hair tips. This same activity was described for *A. tumefaciens* strains 1251 and LBA1010 (Smit et al., 1987, 1989b). Rhicadhesin was further characterized as a small (14 kDa), soluble, extracellular component inactivated by heat and protease treatment (Smit et al., 1989a,b). The gene or genes encoding rhicadhesin have yet to be identified and therefore it is unclear that the rhicadhesin activity isolated from each strain is due to homologous proteins.

An additional set of calcium-binding adhesins were identified in *R. leguminosarum* and *R. etli* in an elegant experiment designed to identify the rhicadhesin coding sequence (Ausmees et al., 2001). Using a phage-display cloning approach the genes for four Rap (*Rhizobium*-adhering proteins) proteins were isolated. The phylogenetic distribution of these proteins is limited compared to rhicadhesin and it is unlikely that they represent the same activity. The Rap proteins were originally proposed to be agglutinins secreted by the PrsD–PrsE type I secretion system. These proteins recognize a polar cell-surface receptor on the bacterium and are capable of mediating autoagglutination and possibly attachment to plant roots, glass, and polystyrene (Russo et al., 2006). Recent work has demonstrated that RapA2 of *R. leguminosarum* specifically binds the acidic exopolysaccharide in a calcium-dependent manner and may contribute to development of the biofilm matrix in this organism (Abdian et al., 2013). No Rap proteins have been identified in *A. tumefaciens* and thus, as for rhicadhesin, any role for these proteins in attachment or biofilm formation by *A. tumefaciens* is speculative.

### THE ROLE OF THE At PLASMID

Initial attempts at isolation and characterization of *A. tumefaciens* mutants that were impaired in early stages of attachment were extensively reported but ultimately raised several questions that have yet to be fully resolved. Tn5 transposon mutagenesis and microscopic observation of mutants unable to attach to carrot suspension culture cells led to the identification of a 29-kb region of genomic DNA that was hypothesized to harbor multiple *att* genes involved in attachment (Matthysse, 1987; Matthysse et al., 2000). At the time of the initial isolation and characterization of the *att* genes the complete genome sequence of *A. tumefaciens* had not been published. The *A. tumefaciens* C58 genome sequence revealed that the *att* genes were located on the accessory plasmid, pAtC58 (Goodner et al., 2001; Wood et al., 2001). This result conflicted with earlier reports that the pAt plasmid was not required for virulence (Hooykaas et al., 1977; Rosenberg and Huguet, 1984; Hynes et al., 1985). It was later confirmed that although the pAt plasmid can mildly influence virulence and ecological fitness of the organism, pAtC58-cured derivatives remain fully virulent with no obvious attachment or virulence deficiency (Nair et al., 2003; Morton et al., 2013). It was further reported that several of the original *att* transposon insertions generated dominant negative alleles and thus the effect of the intact genes was questioned (Matthysse et al., 2008). It seems likely that the pAt plasmid may influence *A. tumefaciens* ecology by broadening the scope of nutritional resources in the rhizosphere via genes that impart catabolism of several common soil compounds (Baek et al., 2005; Chai et al., 2007). Nonetheless, the role of this plasmid and the *att* genes in attachment and biofilm formation, if any, is unclear.

### IMPACT OF THE PLANT HOST ON ATTACHMENT AND BIOFILMS

It is clear that association of bacteria with plant tissues is profoundly, in some cases, dominantly, influenced by the host plant. Nutrient exudation, surface chemistry and defense responses all combine to influence which bacteria efficiently colonize the plant, establishing beneficial, neutral, or pathogenic interactions. In several cases, specific receptors have been identified, such as plant lectins that recognize specific polysaccharides produced by colonizing rhizobia (van Rhijn et al., 2001). There are several candidates for plant surface receptors for *A. tumefaciens*, as well as other plant functions that are required for *A. tumefaciens* infection and T-DNA transfer. Using a collection of T-DNA disruption libraries in the host plant *Arabidopsis thaliana* several candidate plant receptors for *A. tumefaciens* were identified (Gelvin, 2010). These include mutants for an arabinogalactan protein, AtAGP17, a cellulose synthase-like protein, CslA-09, and β-expansin, so-called *rat* mutants (resistant to *Agrobacterium* transformation; Nam et al., 1999; Zhu et al., 2003; Gaspar et al., 2004). Using an analogous screen for *Arabidopsis* mutants that were hypersusceptible to *Agrobacterium* transformation (*hat* mutants) the putative plant receptor protein AT14A was identified as required for efficient attachment (Sardesai et al., 2013). Direct screens for proteins that interact with the Vir machinery also identified potential targets (Hwang and Gelvin, 2004). It remains unclear which of these candidate functions plays a major role in initial attachment, and it is certainly plausible that attachment processes which lead to T-DNA transfer are not identical to those that result in benign associations. There remains much to learn about the bacterial population dynamics on plant tissue surfaces, the impact of plant structures and its response to the colonizing bacteria, and how these influence the outcome of interactions of plants with *A. tumefaciens* in the natural environment.

## REGULATION OF ATTACHMENT AND BIOFILM FORMATION

**FIGURE 2:**
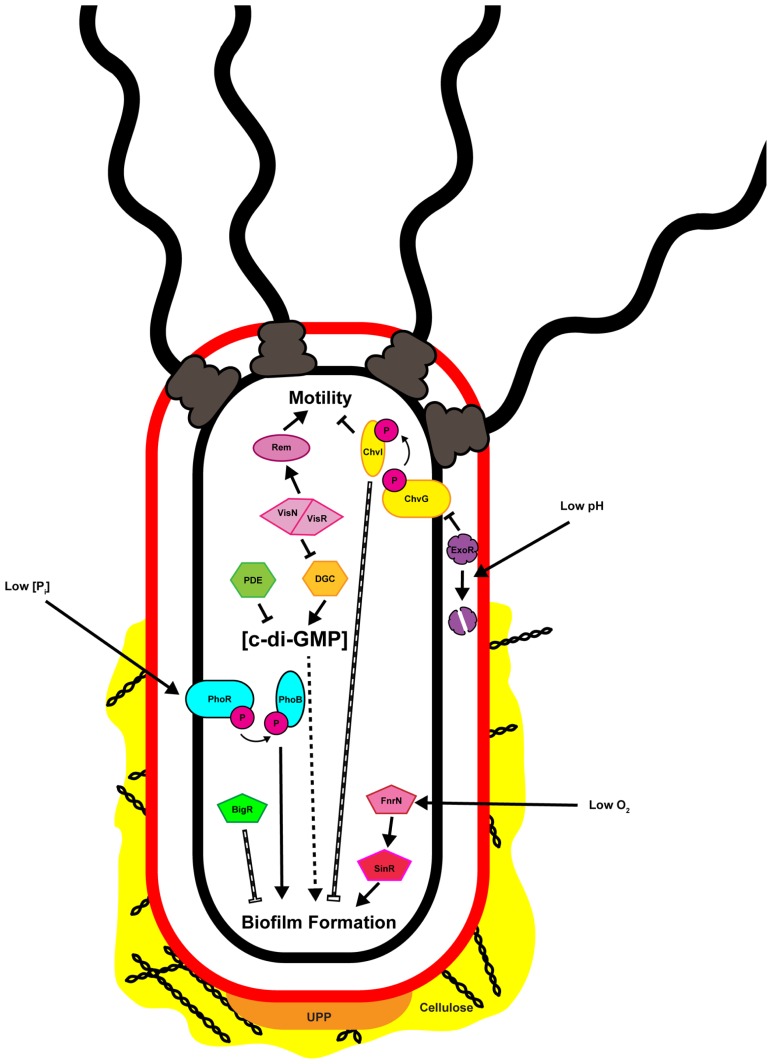
**Multiple inputs regulate attachment and biofilm formation by *Agrobacterium tumefaciens*.** Depicted in the image are the known factors regulating attachment and biofilm formation and discussed in the text. Solid black arrows and bars indicate direct positive or negative regulation, respectively. Hashed arrows and bars indicate regulation that is indirect or where the molecular mechanism has not been defined. Note that the cell envelope is represented only by the outer (red) and inner (black) membranes, and the periplasmic peptidoglycan is not shown.

The transition of bacteria from the motile to the sessile lifestyle, and then to the biofilm mode of growth involves several phenotypic changes mediated at both transcriptional and post-translational levels. Following initial surface contact, flagellar motility is often repressed post-translationally utilizing mechanisms ranging from rotational slow-down to complete flagellar ejection (Shapiro and Maizel, 1973; Aldridge and Jenal, 1999; Blair et al., 2008). Repression of motility allows for stabilization of surface interactions and irreversible attachment mediated by one or more adhesins (Foster and Hook, 1998; Hinsa et al., 2003; Tsang et al., 2006; Berne et al., 2013; Xu et al., 2013). Once irreversibly attached to a surface individual cells can aggregate, forming microcolonies that become enmeshed by the biofilm matrix (Flemming and Wingender, 2010). Within the biofilm cells may communicate, grow, divide, and die, resulting in a metabolically and developmentally heterogeneous population (Stewart and Franklin, 2008). Although establishment of a biofilm is often considered an irreversible process for an individual bacterium there are occasions when the biofilm matrix is actively degraded resulting in dispersal of embedded cells. While dispersal has been observed for attached and biofilm-associated *A. tumefaciens* the mechanism by which this occurs, and how it is regulated, has not been described (Hibbing and Fuqua, 2012). Surface contact, environmental conditions such as oxygen and phosphate levels and pH, and intracellular signaling molecules, often integrated through transcriptional regulatory pathways or posttranscriptional controls have been shown to directly influence attachment and biofilm formation in *A. tumefaciens* (**Figure 2**).

### CYCLIC-DI-GMP

**FIGURE 3:**
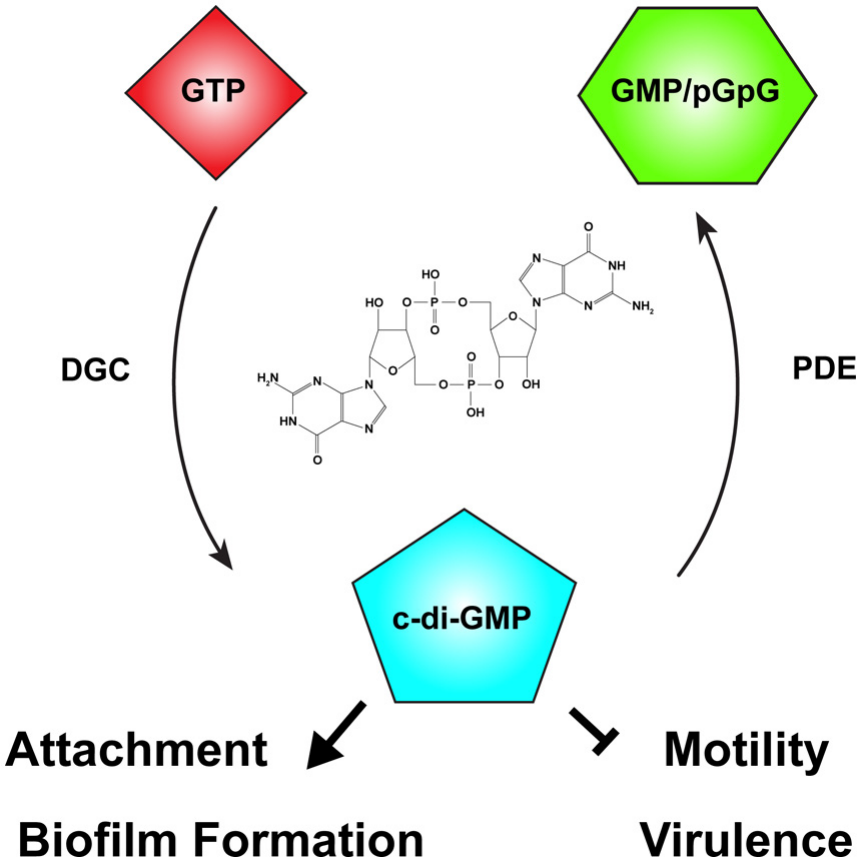
**The second messenger cyclic-di-GMP.** Cyclic diguanylate monophosphate, or cyclic-di-GMP (c-di-GMP) is a common second messenger in prokaryotic systems. C-di-GMP is generated from two molecules of guanosine triphosphate (GTP) by diguanylate cyclases (DGC) and degraded by phosphodiesterases (PDE) to the linear form, 5′-phosphoguanylyl-guanosine (pGpG), and ultimately to two molecules of guanosine monophosphate (GMP). In many bacteria, including *Agrobacterium tumefaciens*, c-di-GMP levels reciprocally regulate the transition between motility and attachment. In *A. tumefaciens* globally or locally increased c-di-GMP levels positively regulate attachment and biofilm formation while negatively regulating motility. The effect of c-di-GMP on virulence in *A. tumefaciens* has not been described, although in many bacteria virulence is negatively regulated by elevated c-di-GMP levels. The chemical structure of c-di-GMP is included in the center of the figure.

One of the primary signaling molecules that controls the motile-to-sessile transition in diverse bacteria is now recognized to be c-di-GMP (**Figure 3**; Hengge, 2009; Römling et al., 2013). Cyclic nucleotides are widespread in both prokaryotes and eukaryotes, with phenotypic effects ranging from nutrient utilization and cell division (cAMP), to cyst formation and pathogenesis (cGMP), to cell cycle control (c-di-AMP; Botsford and Harman, 1992; Beavo and Brunton, 2002; Witte et al., 2008; Gomelsky, 2011; Marden et al., 2011; An et al., 2013). C-di-GMP was first described as a molecule that could activate cellulose synthase in *G. xylinus* and *A. tumefaciens* (Ross et al., 1987; Amikam and Benziman, 1989). Over two decades of research has discovered a variety of bacterial phenotypes regulated by c-di-GMP, including biofilm formation, cell cycle progression, and motility, among others (Römling et al., 2013).

The intracellular concentration of c-di-GMP is controlled by the opposing action of two enzymatic functions: diguanylate cyclases (DGCs), that synthesize c-di-GMP from two molecules of the common nucleotide GTP, and phosphodiesterases (PDEs), that degrade it (**Figure 3**; Schirmer and Jenal, 2009). DGC proteins are characterized by a GGDEF catalytic motif (Paul et al., 2004). Many DGCs also contain an allosteric inhibitory region known as the I-site (Chan et al., 2004). C-di-GMP-specific PDEs are characterized by the presence of either an EAL or HD-GYP catalytic motif (Schmidt et al., 2005; Ryan et al., 2006; Rao et al., 2008). The ubiquity of c-di-GMP signaling was evident early on, with GGDEF- and EAL-containing domains recognized as conserved domains of unknown function (DUF1 and DUF2, respectively) prior to demonstration of their enzymatic activity. Many bacteria have multiple proteins with GGDEF and EAL domains, often associated with other regulatory domains. Many proteins also have both DGC and EAL domains, and the same protein may catalyze c-di-GMP synthesis and degradation. Each domain can be individually regulated, hinting at the complexity and diversity of c-di-GMP-specific signaling. C-di-GMP generally functions allosterically by binding to regulatory domains in proteins or RNA molecules. There are several common c-di-GMP-binding domains found in bacteria including the PilZ domain, at least one two-component response regulator, degenerate (non-functional) EAL domains, and I-sites proximal to inactive GGDEF domains. Binding of c-di-GMP to these domains may be transduced to *cis* regulatory domains within the same protein or to *trans* signal transduction partners that ultimately effect a c-di-GMP-dependent phenotype (Pratt et al., 2007; Römling et al., 2013). Several transcription factors are c-di-GMP responsive, transducing the signal to changes in gene expression (Hickman and Harwood, 2008; Leduc and Roberts, 2009; Krasteva et al., 2010). In addition, riboswitches that specifically sense c-di-GMP with extremely high affinity (K_D_ ~ 1 nM) have been shown to modulate transcriptional activity and RNA splicing (Sudarsan et al., 2008; Lee et al., 2010).

Although c-di-GMP can control a wide range of phenotypes, a common regulatory pattern of c-di-GMP signaling entails altered levels reciprocally affecting two primary phenotypes: motility and attachment. Increasing c-di-GMP levels generally leads to reduced motility and concomitant enhanced attachment. Examples of c-di-GMP-dependent motility phenotypes include the complete flagellar ejection seen in *C. crescentus*, and the reduction of swimming velocity by interaction of a c-di-GMP binding protein with the flagellar motor, observed for *E. coli* (Aldridge and Jenal, 1999; Boehm et al., 2010). C-di-GMP levels may affect both adhesin production and maintenance of these adhesins on the cell surface. This is demonstrated by control of secretion of MRP adhesin in *Pectobacterium atrosepticum* and preservation of the LapA adhesin on the *Pseudomonas fluorescens* cell surface (Newell et al., 2011; Perez-Mendoza et al., 2011). In addition, production of biofilm matrix components is often influenced by c-di-GMP. A recent example is the allosteric control of poly-β-1-6-*N*-acetylglucosamine (poly-GlcNAc) synthesis and secretion in *E. coli* by direct allosteric control of the biosynthetic enzyme complex by c-di-GMP (Steiner et al., 2013). Finally, virulence can be modulated by c-di-GMP signaling, as seen in *Y. pestis* and *V. cholerae* (Pratt et al., 2007; Bobrov et al., 2011).

*Agrobacterium tumefaciens* possesses 33 proteins predicted to be involved in modulating intracellular levels of c-di-GMP (16 GGDEF, 1 EAL, 1 HD-GYP, 13 GGDEF-EAL). This large number of proteins likely reflects the importance of c-di-GMP signaling in the control of *A. tumefaciens* phenotypes. One *A. tumefaciens* phenotype influenced by c-di-GMP was recognized early on with the observation that cellulose synthase activity in crude extracts increased upon the addition of micromolar levels of c-di-GMP (Amikam and Benziman, 1989). It followed from this observation that cellulose-dependent attachment to plant surfaces was also likely influenced by c-di-GMP levels. Ectopic expression of the wild-type *A. tumefaciens* PleD (homologous to *C. crescentus* PleD, the first characterized GGDEF DGC protein) artificially elevated the intracellular levels of c-di-GMP, resulting in a drastic increase in both cellulose and UPP production (Paul et al., 2004; Xu et al., 2013). Increased production of cellulose and UPP coincided with enhanced cellulose-dependent aggregation, UPP-dependent rosette formation, attachment to glass and PVC coverslips, and biofilm formation. C-di-GMP signaling in *A. tumefaciens* appears to follow the paradigm of inverse regulation of motility and attachment as reduced motility was also observed upon c-di-GMP elevation (Xu et al., 2013).

Several activities are regulated by c-di-GMP in *A. tumefaciens*, but it remains unclear how the activity of the various DGCs and PDEs is controlled and how this control is integrated with the motile-to-sessile switch and the production of adhesive polysaccharides. Recently, it was suggested that increased attachment under conditions of limiting phosphate was mediated, at least in part, by a PhoB-dependent increase in c-di-GMP levels (Xu et al., 2012). Thus, environmental conditions seem likely to contribute to regulation of DGC and PDE activity. Transposon mutagenesis of a strain engineered to lack all known exopolysaccharides except UPP identified several mutants with increased UPP production. Of particular interest are four genetic loci in which multiple transposon mutants were isolated (Xu et al., 2013). These loci include two LuxR-type transcription factors (*visN* and *visR*), a CheY-type single domain response regulator (*rrpX*), a putative short-chain dehydrogenase/pteridine reductase (*pruA*), and a dual GGDEF-EAL protein. Further analysis of the role of VisN and VisR identified three DGC homologs that are regulated through VisNR.

### VisN/VisR

VisN and VisR are members of the LuxR–FixJ family of transcriptional regulators that play a critical role in regulating motility in several members of the Rhizobiaceae, including *A. tumefaciens* (Sourjik et al., 2000; Xu et al., 2013). VisN and VisR were first identified as global regulators of motility in *S. meliloti* (Sourjik et al., 2000). The C-termini of both VisN and VisR show strong homology to the DNA-binding domain of LuxR. The N-termini, however, share little homology either with one another or with other known LuxR-family transcriptional regulators, although these N-terminal domains are conserved among orthologs within the Rhizobiaceae. VisN and VisR are believed to function together to regulate transcription of chemotaxis and flagellar motility genes in *S. meliloti*, presumably forming heteromultimers (Sourjik et al., 2000; Rotter et al., 2006; Xu et al., 2013).

As mentioned above, VisN and VisR were originally identified as negative regulators of UPP synthesis and, consequently, attachment and biofilm formation (Xu et al., 2013). Mutations in either *visN* or *visR* also result in a loss of motility in *A. tumefaciens*, consistent with their role as positive regulators of motility in *S. meliloti* and *R. leguminosarum* (Sourjik et al., 2000; Tambalo et al., 2010; Xu et al., 2013). Inverse regulation of motility and biofilm formation by VisNR resembles c-di-GMP-dependent regulation of these same phenotypes in *A. tumefaciens*. Phenotypic and transcriptomic analysis identified three DGCs, *dgcA*, *dgcB*, and *dgcC*, as components of the VisNR regulatory network (Xu et al., 2013). Curiously, deletion of *dgcA*, *dgcB*, or *dgcC*, alone or in any combination does not affect average cytoplasmic levels of c-di-GMP in *A. tumefaciens* cells. This observation supports models where local pools of c-di-GMP and c-di-GMP-dependent effectors play a more defined role in regulating developmental phenotypes, over and above mean cytosolic concentration.

Microarray analysis of the VisNR regulon identified *dgcB* and *dgcC* as transcriptionally regulated by VisNR. DgcA, which plays the dominant role in VisNR-dependent regulation of biofilm formation, was not recognized to be transcriptionally regulated by VisNR. Similarly, microarray analysis of a positive regulator of attachment, ExoR (described below), does not reveal any obvious candidates for transcriptionally controlled regulators of biofilm formation, with the exception of a number of uncharacterized DGC genes (Heckel et al., in review). These observations suggest that control of biofilm formation through the VisNR and ExoR regulons proceeds primarily through post-transcriptional mechanisms. Two other classes of genes are commonly regulated by VisNR and ExoR: the *exo* genes controlling succinoglycan biosynthesis and the *imp* genes controlling type VI secretion (Wu et al., 2012; Xu et al., 2013; Heckel et al., in review). Both of these gene groups, however, are oppositely regulated by VisNR and ExoR. The *exo* and *imp* genes display reduced expression in a Δ*visR* mutant and enhanced expression in Δ*exoR* strains, suggesting positive regulation by VisNR and repression by ExoR (Heckel et al., in review).

### ExoR-ChvG/ChvI

The periplasmic regulator ExoR is a positive regulator of attachment and biofilm formation in *A. tumefaciens* (Tomlinson et al., 2010). ExoR was originally described as a repressor of exopolysaccharide synthesis in *S. meliloti* (Doherty et al., 1988). Additional phenotypes affected in *S. meliloti exoR* mutants include increased biofilm formation, reduced motility, loss of prototrophy, and reduced symbiotic efficiency (Yao et al., 2004; Wells et al., 2007). Several of these phenotypes are consistent with *A. tumefaciens exoR* mutants, including enhanced production of succinoglycan and reduced motility, although in contrast to *S. meliloti* these mutants exhibit attachment and biofilm defects (Tomlinson et al., 2010; Heckel et al., in review).

ExoR exerts its effects primarily through direct inhibition of the two-component system ChvG/ChvI (**Figure 2**; Wells et al., 2007; Chen et al., 2008; Wu et al., 2012; Heckel et al., in review). The ChvG/ChvI two-component system, homologous to ExoS/ChvI of *S. meliloti*, is an acid-responsive signaling system required for virulence (Charles and Nester, 1993; Mantis and Winans, 1993; Li et al., 2002). A genetic interaction between ExoR and ExoS (ChvG) was originally identified in *S. meliloti* (Doherty et al., 1988; Fujishige et al., 2006; Wells et al., 2007). Direct interaction between periplasmic ExoR and the periplasmic portion of the ExoS (ChvG) histidine kinase was eventually demonstrated for both *S. meliloti* and *A. tumefaciens* (Chen et al., 2008; Wu et al., 2012). Under neutral conditions ExoR represses activity of ExoS (ChvG), and through this interaction also negatively regulates the DNA-binding activity of the ChvI response regulator. Upon acidification of the periplasm ExoR is degraded by an unidentified protease, derepressing ExoS (ChvG) activity, resulting in phosphorylation of ChvI and transcriptional activation of several ChvI-regulated genes (Chen et al., 2008; Lu et al., 2012; Wu et al., 2012). The ExoR-ChvG/ChvI signaling trio is well-conserved among the Rhizobiales, and is responsive to environmental signals relevant to the ecology of the individual organism. For example, in the intracellular mammalian pathogen *Bartonella henselae* BatS/BatR, homologous to ChvG/ChvI, is activated at a pH of 7.4, the physiological pH of mammalian blood (Quebatte et al., 2010). For *A. tumefaciens*, low pH is a virulence-inducing signal that is common to the rhizosphere, allowing the ExoR-ChvG/ChvI system to play a distinct role in the ability of the bacteria to sense and respond to potential host plants (Winans, 2008).

Although ExoR-ChvG/ChvI activity and regulation in *A. tumefaciens* is quite similar to that in *S. meliloti* there are two important differences. First, in *A. tumefaciens* mutations in this pathway dramatically diminish attachment and biofilm formation, whereas in *S. meliloti* these mutations enhanced biofilm formation (Fujishige et al., 2006; Tomlinson et al., 2010). Second, while *exoR* is readily deleted from the genome of *A. tumefaciens*, it has been historically difficult to obtain such a mutant in *S. meliloti*. This suggests that control of this important regulatory circuit has diverged in these lineages, perhaps to support the commensal lifestyle of *S. meliloti* and pathogenicity in *A. tumefaciens*, respectively.

### ENVIRONMENTAL AND NUTRITIONAL INPUTS

As with other bacteria, *A. tumefaciens* is responsive to local environmental conditions. As discussed below, efficient induction of the virulence genes of the tumor-inducing (Ti) plasmid occurs under conditions that mimic those found in the plant host rhizosphere. These conditions include low pH and limiting phosphate concentrations. Full virulence induction also requires the presence of plant phenolics such as acetosyringone. The integration of the virulence response with environmental conditions allows for expression of the full suite of virulence genes to occur at a location most likely to result in a productive host–pathogen interaction. Attachment and biofilm formation are also responsive to local environmental conditions. Within and around a microbial biofilm there are expected to be differing environmental conditions such as gradients of oxygen tension, redox potential, and metabolites (Stewart and Franklin, 2008; Koley et al., 2011). Multiple environmental and nutritional inputs have been shown to regulate attachment and biofilm formation by *A. tumefaciens*, including oxygen levels and phosphate concentrations. The pH impacts attachment and biofilm formation through the ExoR-ChvG/ChvI regulatory pathway described above (**Figure 2**). Oxygen tension is proposed to affect biofilm maturation through two independent regulatory pathways, SinR/FnrN and BigR (biofilm growth-associated repressor), both of which are described further below (**Figure 2**).

#### Phosphorus levels and biofilm formation

In *S. meliloti* the production of two exopolysaccharides, EPS I (succinoglycan) and EPS II (galactoglucan), is differentially regulated by phosphate concentration (Rinaudi et al., 2006; Rinaudi and Giordano, 2010). Both of these exopolysaccharides participate in productive biofilm formation in *S. meliloti*, with increased biofilm levels under P_i_ limitation (Rinaudi and Gonzalez, 2009). In *A. tumefaciens* limiting P_i_ levels increase attachment and biofilm formation, an effect that is not succinoglycan-dependent (Danhorn et al., 2004; Tomlinson et al., 2010; Xu et al., 2012). This effect was regulated by the canonical PhoR/PhoB phosphate-sensing two-component system (**Figure 2**). *A. tumefaciens* is unusual in that both the *phoR* and *phoB* genes are essential, under phosphate-replete and phosphate-limiting conditions (Danhorn et al., 2004; Xu et al., 2012). Increased attachment under limiting P_i_ is directly mediated by the UPP adhesive polysaccharide. Interestingly, experimental analysis of the *upp* biosynthetic genes in low phosphate revealed a conditional redundancy for the *uppE* gene, described above (Xu et al., 2012). The effects of P_i_ levels on attachment and biofilm formation have been observed in other Rhizobiaceae, including *R. leguminosarum*, indicating that it may be a conserved response among these bacteria (Janczarek and Skorupska, 2011). However, an inverse relationship between phosphate concentration and biofilm formation is not universal. For example, with *Pseudomonas fluorescens* elevated phosphate levels increased adherence in a PhoR/PhoB-dependent manner and ultimately through c-di-GMP (Monds et al., 2001, 2007).

#### Redox regulation of biofilm formation

As biofilm growth and maturation proceed the local within-biofilm environment experiences several changes, including a reduction in available oxygen, particularly for actively aerobic bacteria (Stewart and Franklin, 2008). In order to survive microaerobic conditions, many bacteria, including *A. tumefaciens*, undertake a respiratory shift from oxic to anoxic conditions, utilizing nitrate rather than oxygen as a terminal electron acceptor (Bueno et al., 2012). In many Alphaproteobacteria, including *A. tumefaciens*, this process, denitrification, is regulated by one or more members of the FNR (fumarate and nitrate reductase) family of transcriptional regulators. *A. tumefaciens* has four such regulators: FixK, FnrN, NnrR, and SinR. Three of these, FnrN, NnrR, and SinR, clearly play a role in regulating denitrification genes in low-oxygen environments, including at the plant interface (Baek et al., 2008). In addition, both SinR and FnrN have been shown to affect biofilm maturation (Ramey et al., 2004b).

The *sinR* locus was initially identified in *A. tumefaciens* during a screen to isolate mutants deficient in biofilm formation (Ramey et al., 2004b). SinR mutants attach and initiate biofilm formation but are deficient in biofilm maturation, never reaching the same structure and cell density achieved by wild-type *A. tumefaciens*. Directly upstream of *sinR* is a canonical FNR-type binding site, and both FnrN and SinR regulate expression of *sinR*. While mutations in FnrN do not display a decrease in biofilm formation Δ*sinR ΔfnrN* double mutants approximate the Δ*sinR* phenotype. Ectopic expression of *sinR* in wild-type, Δ*sinR*, Δ*fnrN*, and Δ*sinR* Δ*fnrN* backgrounds accelerates biofilm maturation and leads to the formation of denser biofilms on both abiotic and plant surfaces (Ramey et al., 2004b).

Oxygen-sensing FNR homologs frequently acquire an oxygen-labile [4Fe–4S]^2^^+^ cluster under low-oxygen conditions, leading to dimerization, DNA binding, and regulation of target genes (Lazazzera et al., 1996). In *A. tumefaciens*, only FnrN is predicted to function in this manner and FnrN upregulates both *sinR* and denitrification genes under low-oxygen conditions (Ramey et al., 2004b; Baek et al., 2008). Together these data suggest that FnrN allows for coordinate regulation of biofilm maturation and respiration under microaerobic or anoxic conditions, allowing *A. tumefaciens* to adjust to local environmental conditions. Although FnrN and SinR both ultimately affect biofilm maturation their regulatory networks are poorly defined, and it is unclear which target genes play a role in biofilm maturation.

BigR is a member of the ArsR/SmtB subfamily of metal-sensing winged-helix transcription factors. In contrast with most members of this family, BigR and its homologs act as redox switches that, upon oxidation, form an intramolecular Cys–Cys disulfide bond. The resulting conformational change reduces the affinity of BigR for its DNA binding site and allows for derepression of the *bigR* operon (Guimaraes et al., 2011). Thus far BigR has been shown to regulate the activity of a single operon, found in both *Xylella fastidiosa* and *A. tumefaciens*, encoding a putative sulfur dioxygenase Blh, BigR itself, and at least three additional putative membrane proteins, one of which likely acts as a sulfite exporter. The *bigR* operon is induced when either *X. fastidiosa* or *A. tumefaciens* is grown as a biofilm on glass coverslips, and a *bigR* mutant generated thicker biofilms on both glass coverslips as well as *Nicotiana tabacum* roots (Barbosa and Benedetti, 2007). It was proposed that detoxification of metabolically generated hydrogen sulfide by Blh would be particularly important under conditions of low oxygen tension such as those found within a biofilm. The mechanism by which BigR would be oxidized in these conditions, thus derepressing transcription of the necessary detoxification genes including *blh*, is not understood, although the authors speculate that hydrogen sulfide-induced reactive oxygen species may play a role (Guimaraes et al., 2011).

## MULTICELLULARITY AND DEVELOPMENT

During its lifetime a single *A. tumefaciens* bacterium must precisely coordinate cell growth and division with current environmental conditions, including whether or not it is entering or exiting the multicellular biofilm mode of growth. It is now recognized that many, if not most, rhizosphere bacteria exist primarily as residents of a single-species or polymicrobial biofilm. Within the rhizosphere *A. tumefaciens* may attach to and form a biofilm on soil particles or at interfaces on the plant host. Participation as a member of a multicellular community, therefore, is a normal and regulated aspect of *A. tumefaciens* biology with important consequences for its ecology.

### A NOVEL FORM OF CELL DIVISION AMONG DIVERSE ALPHAPROTEOBACTERIA

**FIGURE 4:**
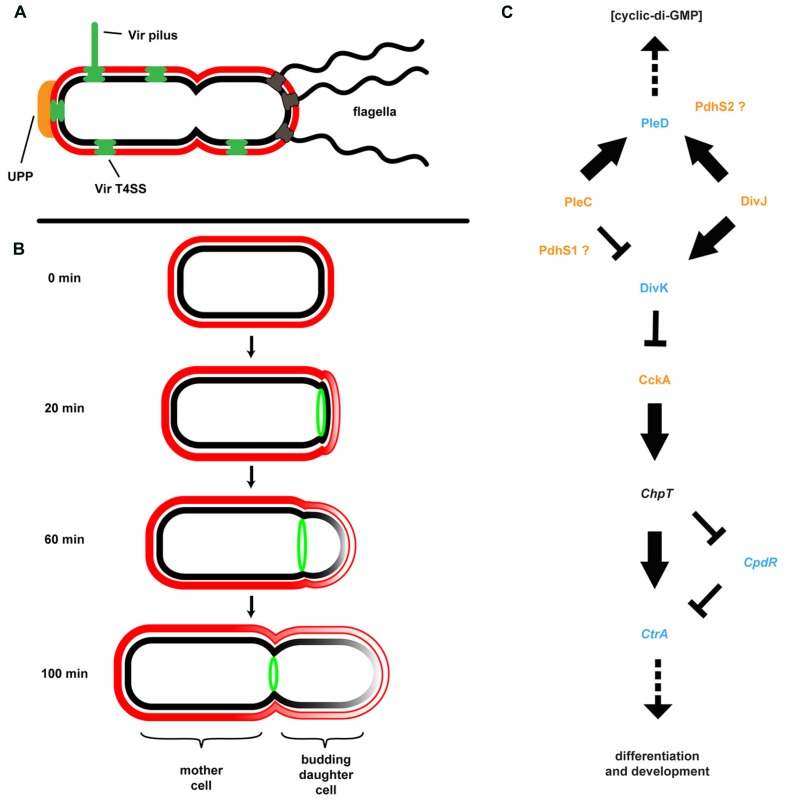
***Agrobacterium tumefaciens* generates and maintains multiple developmental asymmetries. (A)** Shown are morphological features of *A. tumefaciens* known to localize primarily to one pole of the bacterium, including multiple flagella, the unipolar polysaccharide (UPP), Vir pilus and type IV secretion system (T4SS). **(B)** Cell division in *A. tumefaciens* occurs by a polar budding mechanism. The cell division protein FtsZ (green) appears at the site of early constriction, from which the daughter cell buds and at which cytokinesis occurs. The rate of budding indicated is for growth in defined medium (ATGN) on agar pads. **(C)** The coordination of division and development (CDD) regulatory pathway. Proteins for which genetic and/or phenotypic data confirm the suggested pathway architecture are in bold typeface. Italicized proteins are present in the *A. tumefaciens* genome but do not have experimental support; placement of these proteins in the pathway is based on data from other model systems. Histidine kinases are colored orange, response regulators are colored blue, and a single Hpt phosphotransferase (ChpT) is in black text. The location of PdhS1 and PdhS2 is suggested by current data, but not confirmed. Note that the bacterial cell envelope in all panels is depicted as described in **Figure 3**.

Many members of the Rhizobiaceae, including *A. tumefaciens*, are morphological rods and it was presumed that cell division proceeded in much the same way as in the well-studied *E. coli*, *B. subtilis*, and the more closely related Alphaproteobacterium *C. crescentus*. In these model systems division occurs via binary fission. In these systems, individual cells elongate longitudinally by the insertion of new cell wall peptidoglycan and membrane material throughout the length of the cell, followed by septation and cytokinesis. The processes of elongation and septation in these bacteria are directed by conserved protein complexes including the MreB-containing elongase and FtsZ-containing divisome (Margolin, 2009). Other bacteria, such as the Actinobacteria, are known to elongate at the cell poles. In these bacteria pole-directed growth is dependent upon the conserved protein DivIVA and its homologs. Cell growth and division in *A. tumefaciens* and several other Rhizobiales contrasts with both of these known mechanisms for rod-shaped growth. These bacteria lack elongase component homologs as well as DivIVA, but retain one or more copies of FtsZ plus additional divisome components. Time-lapse microscopy coupled with fluorescent protein tracking and selective labeling of outer membrane components detailed a novel budding growth pattern common among *A. tumefaciens*, *S. meliloti*, *Brucella abortus*, *Ochrobactrum anthropi*, and *Hyphomicrobium denitrificans* (Fujiwara and Fukui, 1974; Latch and Margolin, 1997; Brown et al., 2012; Zupan et al., 2013). Budding occurs by insertion of new cell wall and membrane material at a single pole only, followed by septation and cytokinesis (**Figure 4**). Cell division results in two morphologically similar but distinct cell types. One cell, the mother cell, retains old cell wall material while the newly budded daughter cell contains *de novo* synthesized material. Importantly, polar growth was observed in bacteria attached to plant roots with the mother cell attached to the root surface by the UPP and the daughter cell budding into the medium (Brown et al., 2012).

### COORDINATION OF DIVISION AND DEVELOPMENT

Although at first glance it may not be readily apparent, the *A. tumefaciens* life cycle resembles that of the more overtly asymmetric *C. crescentus* (**Figure 4**). *C. crescentus* exhibits a complex, biphasic life cycle that results in the generation of two non-identical cell types: a sessile, non-motile mother cell that often remains attached to a surface, and a motile daughter cell called the swarmer cell (Brown et al., 2009; Curtis and Brun, 2010). The regulatory components underlying this growth, division, and differentiation are well conserved among the Alphaproteobacteria (Brilli et al., 2010). The core architecture of this coordination of division and development (CDD) pathway includes two multicomponent His-Asp phosphorelays converging on multiple response regulators affecting diverse physiological outputs, including c-di-GMP production, motility, biofilm formation, and DNA replication (**Figure 4**). In *C. crescentus*, the master regulator of cell cycle progression is the response regulator CtrA. CtrA directly binds DNA and both blocks replication initiation and affects transcription of multiple target genes. CtrA activity is modulated by phosphorylation and proteolysis via the CckA/ChpT phosphorelay. Activity of the CckA hybrid histidine kinase is, in turn, modulated by the single-domain response regulator DivK. Phosphorylation and dephosphorylation of DivK is mediated by the PdhS family of histidine kinases. These kinases include PleC and DivJ in *C. crescentus* plus additional PleC/DivJ homolog sensor kinases in other bacteria (Hallez et al., 2004, 2007; Pini et al., 2013). *A. tumefaciens* encodes four PdhS proteins: PleC, DivJ, PdhS1, and PdhS2. While several CDD components are essential in *A. tumefaciens*, deletion of many of the non-essential components (PleC, PdhS1, PdhS2, and DivK) affected biofilm formation. Loss of PleC, PdhS1, or DivK disrupted biofilm formation. In contrast mutation of *pdhS2* increased attachment and biofilm formation. These data indicate that the ability to attach to a surface and form a biofilm is integrated into the overall cell cycle program of *A. tumefaciens* (Kim et al., 2013). One mechanism by which this may be achieved is through the response regulator PleD. As described above, PleD is one of several DGCs in *A. tumefaciens* responsible for biosynthesis of the second messenger c-di-GMP (see Cyclic-di-GMP). The activity of PleD is regulated by phosphorylation. In *C. crescentus* and *S. meliloti*, and likely in *A. tumefaciens*, the histidine kinases interacting with PleD are the PdhS family members (Curtis and Brun, 2010; Pini et al., 2013; Sadowski et al., 2013). Deletion of PleD results in a moderate increase in biofilm formation and attachment, although there are other DGCs that appear to have more profound effects (Xu et al., 2013). A complete understanding of CDD regulation of these processes, the effectors, and the molecular mechanisms involved awaits full elucidation (Barnhart et al., 2013, 2014; Kim et al., 2013).

### VIRULENCE

Though studying the motile-to-sessile transition is illuminating in and of itself for understanding bacterial development, it is critical to keep in mind the role that this transition may play as part of the pathogenic lifestyle of *A. tumefaciens*. Virulence of *A. tumefaciens* is mediated by the Ti plasmid, a part of which, called the T-DNA, is translocated into plant host cells and integrated into the host genome to cause tumor formation (Watson et al., 1975; Chilton et al., 1977; Leemans et al., 1981). A critical part of the *Agrobacterium*–plant interaction is attachment of the bacterial cell to a host plant cell, followed by translocation of the T-DNA via a type IV secretion apparatus that spans the bacterial cell wall and somehow provides access to plant cell cytoplasm (Lippincott and Lippincott, 1969; Beijersbergen et al., 1992, 1994). Although attachment to plant tissue frequently leads to biofilm formation, it is clear that in laboratory conditions, biofilm formation is not required for T-DNA transfer (Escudero and Hohn, 1997; Ramey et al., 2004a; Brencic et al., 2005). However, T-DNA transfer is notably inefficient, and attached *A. tumefaciens* cells may be subject to the plant defense response (Veena et al., 2003; Zipfel and Felix, 2005; Zipfel et al., 2006). In natural infections, the large, concentrated population of *A. tumefaciens* cells within a biofilm that forms at a potential infection site may help to overcome these barriers and promote the overall likelihood of a successful T-DNA transfer. Though dense bacterial populations may not be required for virulence *per se*, they are required for pTi maintenance and conjugal dissemination within populations of *A. tumefaciens* associated with infected plants (Fuqua and Winans, 1994).

Biofilms in plant tumors would provide an optimal environment for pTi conjugation, assuring maintenance of the plasmid – and the capacity for infection – among populations of *A. tumefaciens*. There exists an additional relationship between biofilm formation and virulence. In low-phosphate environments, such as in the rhizosphere, both biofilm formation and virulence gene expression are enhanced in *A. tumefaciens* (Winans, 1990; Danhorn et al., 2004). As described above, the phosphate-sensing two-component system PhoR/PhoB mediates an enhanced adherence phenotype, while the pTi-encoded two-component system VirA/VirG mediates the virulence response (Winans, 1990; Danhorn et al., 2004). These regulatory systems potentially work in parallel to allow *A. tumefaciens* cells to attach to plant cells and express virulence genes in a timely manner.

### DISPERSAL

The final “step” in the life of a biofilm is dispersal of members of the microbial community away from the site of attachment and into the environment (**Figure 1**). The ability to inhibit biofilm formation, dissociate the biofilm matrix, or induce active dispersal of the biofilm community is economically, ecologically, and medically relevant. There are multiple known activators of biofilm dispersal in diverse bacteria, including quorum sensing, production of small molecules such as nitric oxide, and secretion of matrix-degrading exoenzymes such as the glycoside hydrolase dispersin or nucleases (McDougald et al., 2012). The D enantiomers of amino acids have also been implicated in biofilm dispersal, although this may be due to indirect effects on protein synthesis (Cava et al., 2011; Leiman et al., 2013). Departure of motile daughter cells away from the attached mother cell upon septation may also serve as a coordinated aspect of biofilm development.

Although dispersal of individual cells from a mature biofilm is proposed to occur at some point in the lifetime of most, if not all, of these multicellular communities, there are few experimental details for this activity in the Rhizobiaceae, including *A. tumefaciens*. Dispersal of *R. leguminosarum* biofilms on abiotic surfaces has been observed but the regulation and mechanism of dispersal, and relevance to surface association with the plant host, have not been defined (Russo et al., 2006). In *A. tumefaciens* the addition of cell-free *P. aeruginosa* culture supernatant stimulated dispersal, although the identity of the active compound secreted by *P. aeruginosa* was not identified (An et al., 2006; Hibbing and Fuqua, 2012). These data suggest that regulated dispersal may be a component of the normal developmental program in *A. tumefaciens*.

## CONCLUSIONS, FUTURE DIRECTIONS, OUTSTANDING QUESTIONS

It is clear that *A. tumefaciens* actively associates with a variety of surfaces in the environment, including but not restricted to those associated with plant hosts. As a metabolically plastic heterotrophic bacterial species, *A. tumefaciens* and its avirulent, plasmidless relatives can occupy a wide variety of environmental niches, and the ability to productively attach to surfaces and form multicellular biofilms is an important and well developed process under complex regulatory control. The asymmetric polar division process exhibited by *A. tumefaciens* is well suited for cells attached via their poles to surfaces in which the mother cell remains sessile and the newly budded daughter cell is released into the environment. Parallels with the well-studied biphasic life cycle of *C. crescentus* are instructive and have led to numerous insights into *A. tumefaciens* cell biology. The molecular targeting mechanisms that lead to polar localization and attachment, along with their coordination, are areas under active study. The orchestration of cell division with the assignment of specific functions to the old pole of the cell or the newer pole created with each round of cell division is a natural extension of such studies. How cytoplasmic c-di-GMP pools are modulated during the transition of motile cells to a sessile state, and the mechanisms by which this is linked to surface contact remain to be discovered. These processes are relevant to *A. tumefaciens* whether or not it is associated with host plants. In the context of plants, *A. tumefaciens* has evolved remarkable mechanisms for colonizing and manipulating its host, most notably culminating in interkingdom gene transfer, neoplastic growth and opine production. It remains unknown how the attachment and biofilm formation mechanisms that are the primary focus of this review are integrated with the events leading to T-DNA transfer. Mutants that are severely hampered in attachment remain virulent as measured using *in vitro* plant inoculation assays. It is unclear whether this is a limitation of these assays, or whether the events and processes leading to T-DNA transfer are truly distinct from those which mediate general surface attachment and subsequent biofilm formation. One plausible explanation is that in the natural environment, there is a temporal progression from general surface attachment, to the induction of *vir* genes and elaboration of the type IV secretion system, plus whatever additional intimate interactions with the plant cells are driven by these functions (including the potential shift to lateral association), and eventual T-DNA transfer. What is required to evaluate this hypothesis is the ability to follow the process from tissue colonization through T-DNA transfer in real time. As yet the tools and approaches for such dynamic monitoring have not been applied to this process, but such a high resolution view of *A. tumefaciens* interactions with plant hosts is a goal for future research.

## Conflict of Interest Statement

The authors declare that the research was conducted in the absence of any commercial or financial relationships that could be construed as a potential conflict of interest.
